# Long non-coding RNA RPPH1 promotes the proliferation, invasion and migration of human acute myeloid leukemia cells through down-regulating miR-330-5p expression

**DOI:** 10.17179/excli2019-1686

**Published:** 2019-09-11

**Authors:** Bo Lei, Aili He, Yinxia Chen, Xingmei Cao, Pengyu Zhang, Jie Liu, Xiaorong Ma, Lu Qian, Wanggang Zhang

**Affiliations:** 1 Department of Hematology, second Affiliated Hospital of Xi'an Jiaotong University,157 Xiwu Road, Xi'an, Shaanxi, China

**Keywords:** long non-coding RNA, RPPH1, acute myeloid leukemia, miR-330-5p

## Abstract

Multiple studies have revealed that the long non-coding RNA RPPH1 (Ribonuclease P RNA Component H1) is involved in disease progression of solid tumors and neurodegenerative diseases. We aimed to explore the functions of RPPH1 in the pathogenesis of acute myeloid leukemia (AML) and the underlying molecular mechanisms. The expression of RPPH1 was examined in blood samples of AML patients and human AML cell lines including THP-1 and HL-60. The microRNAs (miRNAs) targets of RPPH1 were predicted with online tools and validated with the dual luciferase reporter assay. The malignant behaviors of AML cells with lentivirus medicated knockdown of RPPH1 and/or administration of miR-330-5p inhibitor were assessed. Cell proliferation was determined by the CCK-8 and EdU incorporation methods, and cell invasion and migration were assayed with transwell experiments. The effects of RPPH1 knockdown on *in vivo* tumor growth were evaluated in nude mice with xenografted THP-1 cells. RPPH1 was expressed in the AML tissues and cell lines and its high expression predicted worse overall survival in AML patients. miR-330-5p was validated to be a direct target of RPPH1. Knockdown of RPPH1 suppressed the proliferation, invasion and migration ability of human AML cells, which was partially reversed by additional administration with miR-330-5p inhibitor. RPPH1 knockdown significantly inhibited the growth of xenografted THP-1 tumor in nude mice. Our work highlights the contributions of RPPH1 in promoting AML progression through targeting miR-330-5p, and suggests that the RPPH1/miR-330-5p axis is a potential target for AML treatments.

## Introduction

Acute myeloid leukemia (AML) is a malignant clonal disorder of blood cells, which is characterized by alterations and low production of healthy hematopoietic cells due to the expansion of malignant tumor (Dohner et al., 2015[8]; Prada-Arismendy et al., 2017[22]). AML is the most common form of acute leukemia in adults with an incidence of approximately 2-4 cases per 100,000 population per year (Juliusson et al., 2012[14]). AML can be a primary disease, or secondary to hematological disorders or after chemotherapy and/or radiotherapy for another primary disease (Dohner et al., 2015[8]; Schulz et al., 2012[23]). Due to the complexity of molecular and cytogenetic architecture in AML, the exact molecular basis of AML in humans remains not fully elucidated. For a long period of time, the treatment options for AML are limited to chemotherapy and hematopoietic stem cell transplantation (Liao et al., 2019[16]). However, current high-intensity treatment approaches allow around 40 % cure rate in younger AML patients and up to 15 % long lasting remissions in older patients over 60 years of age (Dohner et al., 2010[7]). The efficacy of current AML therapies is limited due to a bunch of problems, such as the occurrence of chemotherapy resistance, intolerance of high-intensity chemotherapy in the elderly, and relapse after transplantation. Therefore, it is urgent to explore novel targets for more effective therapy of AML.

Recently, the development of high-throughput screening technologies has facilitated the identification of the critical roles of numerous non-coding genes, such as long non-coding RNA (lncRNA) and microRNAs (miRNAs) in the pathogenesis of hematological malignancies (Cruz-Miranda et al., 2019[6]; Liu et al., 2019[19]; Wei and Wang, 2015[28]). LncRNA is defined as an RNA transcript longer than 200 nucleotides with no protein product, and was considered to be non-functional accumulation of genome junk sequences (Wapinski and Chang, 2011[27]). However, accumulating evidence suggests that lncRNAs have multiple functions in normal and malignant hematopoiesis (Han and Chen, 2013[12]), and they are widely involved in epigenetic regulation, cell cycle regulation, cell proliferation, cell differentiation, apoptosis, and metastasis (Beltran-Anaya et al., 2016[3]). LncRNAs may regulate gene transcription and translation through different mechanisms, such as interaction with RNA-binding proteins to reduce the translation activity of mRNA and epigenetically regulate gene expression, and competition with mRNAs for miRNA binding. Various lncRNAs were reported to be implicated in the pathogenesis of AML. For instance, lncRNA SBF2-AS1 was recently revealed to modulate cell proliferation of AML cells through acting as a miRNA sponge of miR-188-5p, and SBF2-AS1 inhibition represents a potential therapeutic strategy for AML treatment (Tian et al., 2019[26]). It is also anticipated that lncRNAs will be used in clinical diagnosis and treatment after large scale clinical trials and functional studies are completed in the near future (Feng et al., 2018[10]).

As one of the lncRNAs, RPPH1 (Ribonuclease P RNA Component H1) is well-known as an RNA subunit of RNase P, which participates in the cleaveages of tRNA precursor molecules and formation of the mature 5′termini of the tRNA sequences (Baer et al., 1990[1]). RPPH1 is widely used as an internal housekeeping gene for RNA quantitation (Soler-Alfonso et al., 2014[24]). Nevertheless, recent investigations with deep sequencing technologies have uncovered that RPPH1 is up-regulated in human gastric cancer tissues and neocortical tissues of seizure patients (Lipovich et al., 2012[18]; Xia et al., 2014[29]). Moreover, RPPH1 over-expression was found to promote breast cancer progression through functional suppression of miR-122 and its targets (Zhang and Tang, 2017[32]). Besides, Rpph1 was identified to up-regulate CDC42 expression in mice cortexes and hippocampi through direct binding to miR-326-3p and miR-330-5p. Rpph1 was shown to compete with endogenous miR-330-5p and subsequently up-regulate CDC42 to modulate actin dynamics in primarily cultured pyramidal hippocampal neurons (Cai et al., 2017[5]). Therefore, these reports suggest that RPPH1 is involved in disease progression in animals and humans, but is not merely a “house-keeping enzyme”. However, whether RPPH1 contributes to the pathogenesis of AML and the potential molecular mechanisms are not explored so far.

In this study, we examined the expression level of RPPH1 in blood samples of AML patients and several human AML cell lines, and identified that high RPPH1 expression in AML patients predicted worse overall survival. Through shRNA (short hairpin RNA)-mediated knockdown of RPPH1 expression in AML cells, we revealed the critical roles of RPPH1 in promoting the malignant behaviors of AML cells both *in vitro* and in AML tumor xenograft mice model. In addition, we also explored the interactions between RPPH1 and candidate miRNAs in human AML cells, and confirmed that miR-330-50p was a target miRNA of RPPH1 in AML.

## Materials and Methods

### Cell culture

Human bone marrow stromal cell line HS-5 (ATCC® CRL-11882™), human acute monocytic leukemia cell line (ATCC® TIB-202™), human acute promyelocytic leukemia cell line HL-60 (ATCC® CCL-240™), and human acute myelogenous leukemia cell line KG-1 (ATCC® CCL-246) were originally purchased from the American Type Culture Collection (ATCC, Manassas, VA, USA). These cell lines were authenticated by STR (Short Tandem Repeat) DNA profiling analysis and tested as mycoplasma contamination free by the vender. The base culture medium for HS-5 cells was Dulbecco's Modified Eagle's Medium (DMEM; Thermo Fisher Scientific, Waltham, MA, USA), and that for THP-1 cells was Roswell Park Memorial Institute (RPMI)-1640 (Thermo Fisher Scientific). The base medium for HL-60 cells and KG-1 cells was Iscove's Modified Dulbecco's Medium (IMDM; Thermo Fisher Scientific). To make the complete growth medium, the base medium was supplemented with 10 % fetal bovine serum (FBS; Gibco, Gaithersburg, MD, USA), 100 units/ml of penicillin and 100 µg/ml of streptomycin (Gibco). Cells were cultured in a humidified atmosphere with 5 % CO_2_ at 37 °C.

### Patients 

Patients with AML in second Affiliated Hospital of Xi'an Jiaotong University (Xi'an, China) were enrolled between July, 2018 and May 2019. Peripheral blood sample collections were carried out according to the protocols abided by the Ethics Review Board at second Affiliated Hospital of Xi'an Jiaotong University. Informed consents were signed by all the subjects. Peripheral blood mononuclear cells (PBMCs) were isolated using Ficoll-Paque™ (GE Healthcare Life Sciences, Pittsburgh, PA, USA) through density-gradient centrifigution. 

### shRNA gene knocking down 

Three siRNA sequences and one random negative control sequence (antisense strand sequence:) were cloned into the lentiviral vector. The most effective shRNA sequence (sh-RPPH1-1; antisense strand sequence: 5′-AAGAGUGACACGCACUCAGCACGUG-3′) with a targeting sequence located at the locus of human RPPH1 gene (GenBank Accession No. NR_002312.1) was selected in pre-experiments. Lentiviral particles were produced in HEK 293T cells by transiently co-transfecting control lentiviral vector (sh-NC) or RPPH1-knockdown lentiviral vector (sh-RPPH1) together with helper plasmids pHelper 1.0 (Gag and Pol) and pHelper 2.0 (VSVG) using house-made transfection reagents from GenePharma Co., Ltd (Shanghai, China). The vector constructions, verification by sequencing, virus packaging and collection of the corresponding viral supernatants were performed by GenePharma Co., Ltd (Shanghai, China).

THP-1 or HL-60 cells in logarithmic growth phase were inoculated on 24-well plates at a density of 5×10^4^/ml and cultured for 24 hours. Cells were divided into three groups: experiment group infected with sh-RPPH1, control group infected with sh-NC, and uninfected blank group. Cells were infected by lentivirus with the best Multiplicity of Infection (MOI=70) obtained in the pre-experiments, and the culture medium was changed after 8 hours. Stable cell lines with knockdown of RPPH1 were obtained with the limited dilution method after screening with the antibiotic.

### Cell transfection

The miR-330-5p mimic, the miR-330-5p inhibitor, and negative control (miR-NC) were purchased from Shanghai GenePharma Co., Ltd (Shanghai, China). Transfections of the miRNAs/inhibitor were performed using Lipofectamine 2000 (Thermo Fisher Scientific) according to the manufacturer's instructions. At 48 hours after transfection, cells were subjected to further analyses.

### Real-time RT-PCR

Total RNA was isolated using TRIzol reagent (Invitrogen, Carlsbad, CA) and miRNA was extracted using a miRNeasy mini kit (Qiagen, Hilden, Germany) according to the manufacturer's instructions, and total RNA quality was confirmed by gel electrophoresis. Total RNA (1 μg each sample) was used to synthesize cDNA utilizing the PrimeScript® RT Master Mix Perfect Real Time Reagent Kit (Takara Bio Inc., Shiga Prefecture, Japan). For miRNA reverse transcription, cDNA was synthesized using a universal tag by using a miScript II RT kit (Qiagen). Quantitative reverse transcription PCR (RT-qPCR) for miRNA and mRNA were performed using a standard protocol from the SYBR Green PCR kit (Toyobo, Osaka, Japan) on an AB7500 RT-PCR instrument (Applied Biosystems, Foster City, CA, USA). Relative quantifcation was determined by normalization to GAPDH or U6. PCR primers were synthesized by Sangon Biotech Co. Ltd (Shanghai, China). The PCR reaction protocol consisted of two steps: step one, initial denaturation for 30 s at 95 °C step two, denaturation for 5 s at 95 °C annealing and extension for 31 s at 60 °Cand fluorescence signal acquisition. The reactions had a total of 40 cycles, and ended with a melting curve which consisted of 15 s at 95 °C 1 min at 60 °C 15 s at 95 °C and 15 s at 60 °C. The experiments were repeated for 3 times and each sample was run in triplicates. PCR product specificity was confirmed by melting curve analysis.The primers are listed in the Supplementary Table 1, gene expression levels were calculated with the 2^-ΔΔCT^ method.

### Proliferation assays

THP-1 and HL-60 cell lines with specified manipulation of gene-expression were seeded into 96-well plates at a density of 5 × 10^3^/well. At 24 hours after culturing, the cell viability was evaluated with the Cell Counting Kit-8 (CCK-8; Dojindo Molecular Technologies, Kumamoto, Japan) following the manufacturer's specifications. Briefly, cells were incubated with the CCK-8 reagent at 37 °C for 2 hours. Cell viability was quantified by measuring absorbance at 595 nm on a microplate reader. Cells adherently grown on a coverslip were assayed for cell proliferation with the EdU method (EdU Flow Cytometry Kit 488; Sigma-Aldrich, St. Louis, MO, USA). After fixation with 4 % formaldehyde in phosphate-buffered saline (PBS) and permeabilization with 0.5 % Triton™ X-100 in PBS, cells were stained with xnM 5-Ethynyl-deoxyuridine (5-EdU) and 1 µg/mL DAPI (4′,6-diamidino-2-phenylindole). A Zeiss LSM 700 Meta confocal microscope was used to measure the fluorescence.

### Cell invasion and migration assays

Transwell Boyden chambers (BD Biosciences, San Jose, CA, USA) with 8 μm pore size polycarbonate filters were used for *in vitro* cell migration assays, and chambers coated with Matrigel (BD Biosciences) were used to evaluate the invasive potential of THP-1 and HL-60 cells *in vitro*. Briefly, ES cells were seeded at a density of 1×10^5^ per well into the upper chamber, and 500 μl complete medium was loaded into the lower chamber. Chambers were then incubated for 24 h. At the time of harvesting, cells remaining inside the upper chambers were removed, while migrated and invaded cells on the lower surface of the membrane were fixed in 1 % paraformaldehyde and stained with crystal violet (Sigma-Aldrich), followed by visualization and counting under an inverted microscope. Cells were imaged using a low power (100×) magnification, and five visual fields per well were randomly selected for cell counting.

### Luciferase reporter assay

Candidate miRNAs targeting RPPH1 and the binding sites of miRNAs and RPPH1 were predicted using the online tools starBase v 2.0 (http://starbase.sysu.edu.cn/starbase2/index.php) and lncbase. The predicted RPPH1 mRNA binding site region and the corresponding mutated region were cloned into a luciferase-expressing vector. HEK293 T cells (ATCC® CRL-3216™) were co-transfected with the vectors and the miR-330-5p mimics or scramble control miR-NC. At 48 hours after transfection, the culture supernatant was harvested and subjected to luciferase activity analysis using a Dual-Luciferase Reporter Assay System (Promega, Fitchburg, WI, USA) following the manufacturer's instructions.

### Xenograft tumor model

Four-week-old male BALB/c nude mice were purchased from Charles River Laboratories (Beijing, China), and housed at the specific pathogen-free (SPF) facility at the Animal Center of Xi'an Jiaotong University. Mice were maintained at room temperature (22 ± 1 °C) with a 12/12 hours light/dark cycle and access to food and water *ad libitum*. Stable THP-1 cell line with knockdown of RPPH1 (sh-RPPH1) or control THP-1 cells (sh-NC) were injected subcutaneously into the right flank of each mouse (5×10^6^ cells/mouse) to establish the xenograft model. Tumor volume was monitored twice per week by measuring the tumor diameters, and calculated with the following formula: V=ab^2^/2, where *a* is the long diameter while *b* is the short diameter. Two weeks after tumor inoculation, mice were euthanized and tumor tissues were subjected to further analyses. Animal experiments were conducted in accordance with the Declaration of Helsinki and all procedures involving experimental animals were approved by the University Committee on the Use and Care of Animals (UCUCA) at the second Affiliated Hospital of Xi'an Jiaotong University.

### Statistics

Data are presented as mean ± standard deviation (SD). Statistical analyses were conducted using the SPSS 17.0 software (IBM, Armonk, NY, USA). Student's t-test was used to compare differences between two groups. One-way analysis of variance (ANOVA) was used to analyze differences among more than two groups, which was followed by Tukey's post hoc test. A *P* value less than 0.05 was considered statistically significant.

## Results

### RPPH1 is expressed in the AML tissues and cell lines and its high expression predicts worse overall survival in AML patients

**Figure 1:**
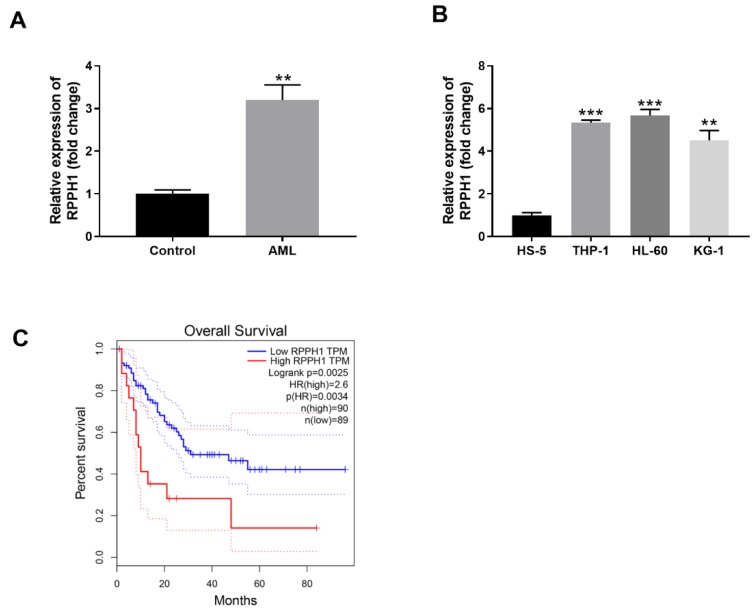
RPPH1 is expressed in AML tissues and cell lines and its high expression predicts worse overall survival of AML patients. (A) Relative expression level of RPPH1 in the PBMCs from healthy control donors (Control) and AML patients (AML) was determined by RT-qPCR. (B) Relative expression level of RPPH1 in human bone marrow stromal cell line HS-5 and three human AML cell lines (THP-1, HL-60 and KG-1) was determined by RT-qPCR. (C) Overall survival analysis by Kaplan-Meier and log-rank test indicated that the AML patients with higher RPPH1 level had worse outcome compared with those with lower RPPH1 level. **P<0.01, compared with the indicated controls

In order to explore the potential roles of lncRNA RPPH1 in AML, we first determined the relative transcript level of lncRNA RPPH1 in the PBMC samples of AML patients and some common AML cell lines. PBMC samples were collected from AML patients and matched healthy control subjects. RT-qPCR analysis demonstrated that RPPH1 expression was significantly higher (approximately 3-fold) in the blood samples of AML group than that in the control group (Figure 1A(Fig. 1)). In addition, compared with the control bone marrow stromal cell line HS-5, three typical AML cell lines including THP-1, HL-60 and KG-1 had significantly higher levels of RPPH1 transcript. While KG-1 cells displayed approximately 4.5 folds expression of RPHH1 in comparison to HS-5, THP-1 and HL-60 cells displayed over 5 folds RPHH1 expression (Figure 1B(Fig. 1)). Therefore, THP-1 and HL-60 cells with more RPPH1 expression were selected for further studies. Moreover, we performed the overall survival analysisby the GEPIA (http://gepia.cancer-pku.cn/detail.php), the results showed that patients with higher RPPH1 expression had worse overall survival than those with lower RPHH1 expression (Figure 1C(Fig. 1)). Taken together, RPPH1 expression is increased in blood cells of AML patients and associated with poor overall survival of AML patients.

### Knockdown of RPPH1 suppressed the proliferation, invasion and migration ability of human AML cells

**Figure 2:**
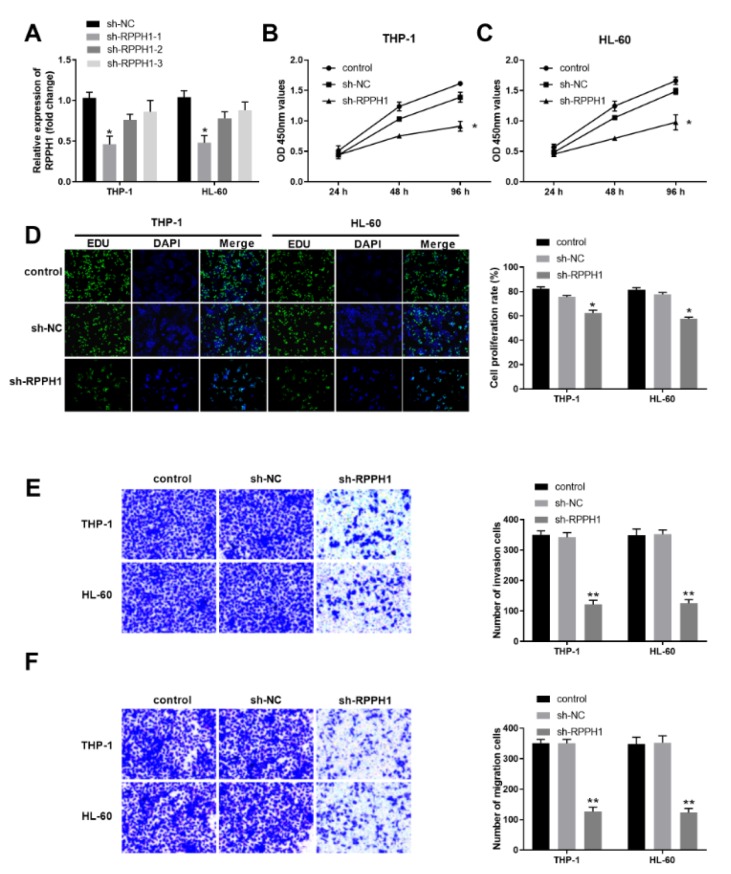
RPPH1 knockdown suppressed the proliferation, invasion and migration ability of THP-1 and HL-60 cells. (A) The relative expression level of RPPH1 in the indicated stable THP-1 and HL-60 cell lines was determined by RT-qPCR. The stable cell lines were established after infection of control lentivirus (sh-NC) or RPPH1-specific shRNA-expressing lentivirus (sh-RPPH1-1, sh-RPPH1-2, and sh-RPPH1-3). (B-C) Cell proliferation in the indicated THP-1 cell lines (B) and HL-60 cell lines (C) was determined with the CCK-8 method. (D) Cell proliferation in the indicated THP-1 and HL-60 cell lines was determined with the EdU method. Representative images show the staining of EdU and DAPI in cells at 48 hours after culturing, and the bar graphs summarize the relative cell proliferation rate, which was determined by the ratio of EdU positive cells to the DAPI positive cells. (E-F) The ability of cell invasion (E) and cell migration (F) of THP-1 and HL-60 cells was determined by the transwell assays. Representative images show the staining of cells at 48 hours after transwell culturing, and the bar graphs summarize the invasion or migration cell numbers. *P<0.05, **P<0.01; compared with the indicated controls

To substantiate the roles of lncRNA RPPH1 in the progression of AML, we determined the effects of RPPH1 knockdown on proliferation, invasion and migration of human AML cell lines THP-1 and HL-60. First, we established three THP-1 cell lines and three HL-60 cell lines with stable knockdown of RPPH1 by shRNA lentivirus infection and screening with the antibiotics.As shown in Figure 2A(Fig. 2), the shRNA construct sh-RPPH1-1 rendered more reduced expression of RPPH1 transcript in THP-1 and HL-60 cells than the other two constructs sh-RPPH1-2 and sh-RPPH1-3. Therefore, cells with sh-RPPH1-1 mediated knockdown of RPPH1 were used for further analysis. Compared with the parental control cells and sh-NC infected cells, either THP-1 cells with reduced RHHP1 expression or HL-60 cells with reduced RHHP1 expression demonstrated significantly decreased proliferation in the CCK-8 assays (Figure 2B and Figure 2C(Fig. 2)) and EdU assays (Figure 2D(Fig. 2)). Furthermore, our transwell assays revealed that RPPH1 knockdown remarkably decreased the invasion ability (Figure 2E(Fig. 2)) and migration ability (Figure 2F(Fig. 2)) of THP-1 cells and HL-60 cells. Collectively, these results suggest that RPPH1 expression contributes to the malignant behaviors of AML cells. 

### The lncRNA RPPH1 and miR-330-5p have a targeting effect relationship in THP-1 cells

**Figure 3:**
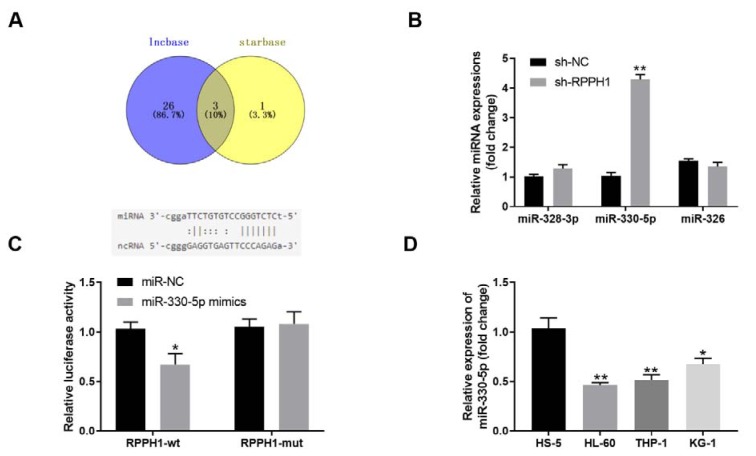
The lncRNA RPPH1 and miR-330-5p have a targeting effect relationship in THP-1 cells. (A) The Venn diagram shows the candidate miRNAs that were predicted to be targeted by the lncRNA RPPH1, which were revealed by two tools starBase v 2.0 and lncbase. Schematic diagram shows the matching base pairs of RPPH1 and miR-330-5p. (B) The relative expression level of indicated miRNAs in the control stable THP-1 cell line (sh-NC) and stable RPPH-knockdown THP-1 cell line (sh-RPPH1) was determined with RT-qPCR. (C)The luciferase activity of the reporter vectors was detected in 293T cells after 48 hour of co-transfection of wild type (RPPH1-wt) or mutant (RPPH1-mut) RPPH1 3'UTR with miR-330-5p mimics. (D) The relative expression level of miR-330-5p in human bone marrow stromal cell line HS-5 and three human AML cell lines (THP-1, HL-60 and KG-1) was determined with RT-qPCR. *P<0.05, **P<0.01; compared with the indicated controls

We then predicted the possible miRNA targets of lncRNA RPPH1 using the tools starBase v 2.0 and lncbase. As shown in Figure 3A(Fig. 3), lncbase revealed 29 candidate miRNA targets and starBase revealed 4 candidate miRNA targets, while 3 miRNAs including miR-328-3p, miR-330-5p, and miR-326 were the common targets of RPPH1 predicted by both tools. Therefore, we focused on these 3 miRNAs and examined their levels in THP-1 cells upon knockdown of RPPH1. Compared with the sh-NC lentivirus infected control cells, the stable THP-1 cell line with sh-RPPH1 lentivirus infection-mediated RPPH1 knockdown had significantly increased expression of miR-330-5p (approximately 4 fold), and largely unchanged expressions of miR-328-3p and miR-326 (Figure 3B(Fig. 3)), which suggested that miR-330-5p was more likely to be regulated by RPPH1. Moreover, the dual luciferase assays demonstrated that the luciferase activities were significantly deceased after co-transfection of wild type RPPH1 (RPPH1-WT) and miR-330-5p mimics, but barely changed after co-transfection of mutated RPPH1 (RPPH-MUT) and miR-330-5p mimics (Figure 3C(Fig. 3)), which implied that RPPH1 regulated the expression of miR-330-5p through directly binding. Furthermore, three human AML cell lines (HL-60, THP-1 and KG-1) with higher RPPH1 expression had markedly reduced expression of miR-330-5p when compared with the control HS-5 cells (Figure 3D(Fig. 3)), which further confirmed that miR-330-5p is a target of RPPH1. Taken together, our results suggest that the lncRNA RPPH1 and miR-330-5p have a targeting effect relationship in THP-1 cells.

### Inhibition of miR-330-5p partially reverses the effects of RPPH1 knockdown on proliferation, invasion and migration ability of human AML cells

**Figure 4:**
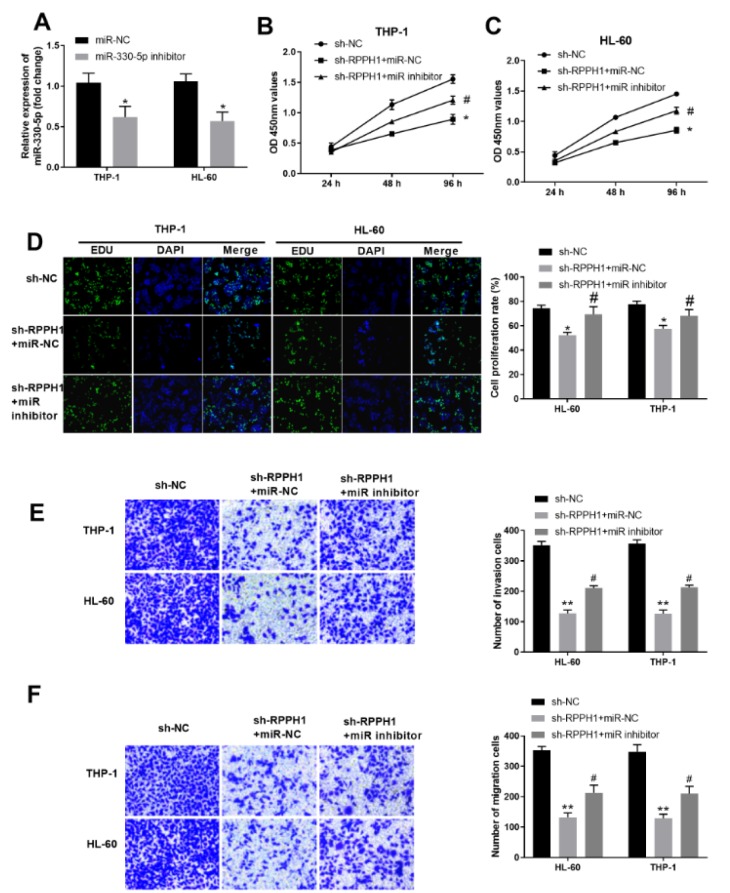
Inhibition of miR-330-5p partially reversed the effects of RPPH1 knockdown on proliferation, invasion and migration ability of THP-1 and HL-60 cells. (A) The relative expression level of miR-330-5p in THP-1 and HL-60 cells transfected with control siR-NC or miR-330-5p inhibitor was determined by RT-qPCR. (B-C) Cell proliferation in the indicated THP-1 cell lines (B) and HL-60 cell lines (C) was determined with the CCK-8 method. RPPH1 knockdown cell lines (sh-RPPH1) were transfected with control miR-NC or miR-330-5p-specific inhibitor (miR-inhibitor), while "shR-NC" denotes the control stable cell line. (D) Cell proliferation in the indicated cell lines was determined with the EdU method. Representative images show the staining of EdU and DAPI in cells at 48 hours after culturing, and the bar graphs summarize the relative cell proliferation rate.(E-F) The ability of cell invasion (E) and cell migration (F) of the indicated cell lines was determined by the transwell assays. Representative images show the staining of cells at 48 hours after transwell culturing, and the bar graphs summarize the invasion or migration cell numbers.*P<0.05, **P<0.01, compared with the indicated sh-NC controls; ^#^*P*<0.05, compared with the "sh-RPPH1+miR-NC" group

Since miR-330-5p was identified as a target of RPPH1, we further explored whether miR-330-5p was involved in the contribution of RPPH1 on proliferation, invasion and migration of AML cells. First, we validated that transfection of miR-330-5p inhibitor significantly reduced the level of miR-330-5p in both THP-1 cells and HL-60 cells (Figure 4A(Fig. 4)). As knockdown of RPPH1 can significantly increase the level of miR-330-5p in AML cells (Figure 3B(Fig. 3)), we then tested the effects of miR-330-5p inhibition on the malignant behaviors of AML cells with reduced RPPH1 expression. Compared with the sh-RPPH1-lentivirus infected stable THP-1 and HL-60 cells with further transfection of control miR-NC, the sh-RPPH1-lentivirus infected stable cells with additional transfection of miR-330-5p-specific inhibitor had increased proliferation ability, as assayed by the CCK-8 method (Figure 4B and Figure 4C(Fig. 4)) and EdU method (Figure 4D(Fig. 4)). Consistently, transfection of miR-330-5p inhibitor also partially reversed the decreased invasion ability (Figure 4E(Fig. 4)) and migration ability (Figure 4F(Fig. 4)) of AML cells with reduced expression of RPPH1. Collectively, these results indicated that miR-330-5p inhibition partially reversed the effects of RPPH1 knockdown in AML cells, and contributed to the malignant behaviors of AML cells.

### Knockdown of RPPH1 inhibits the growth of xenografted THP-1 tumor in nude mice

**Figure 5:**
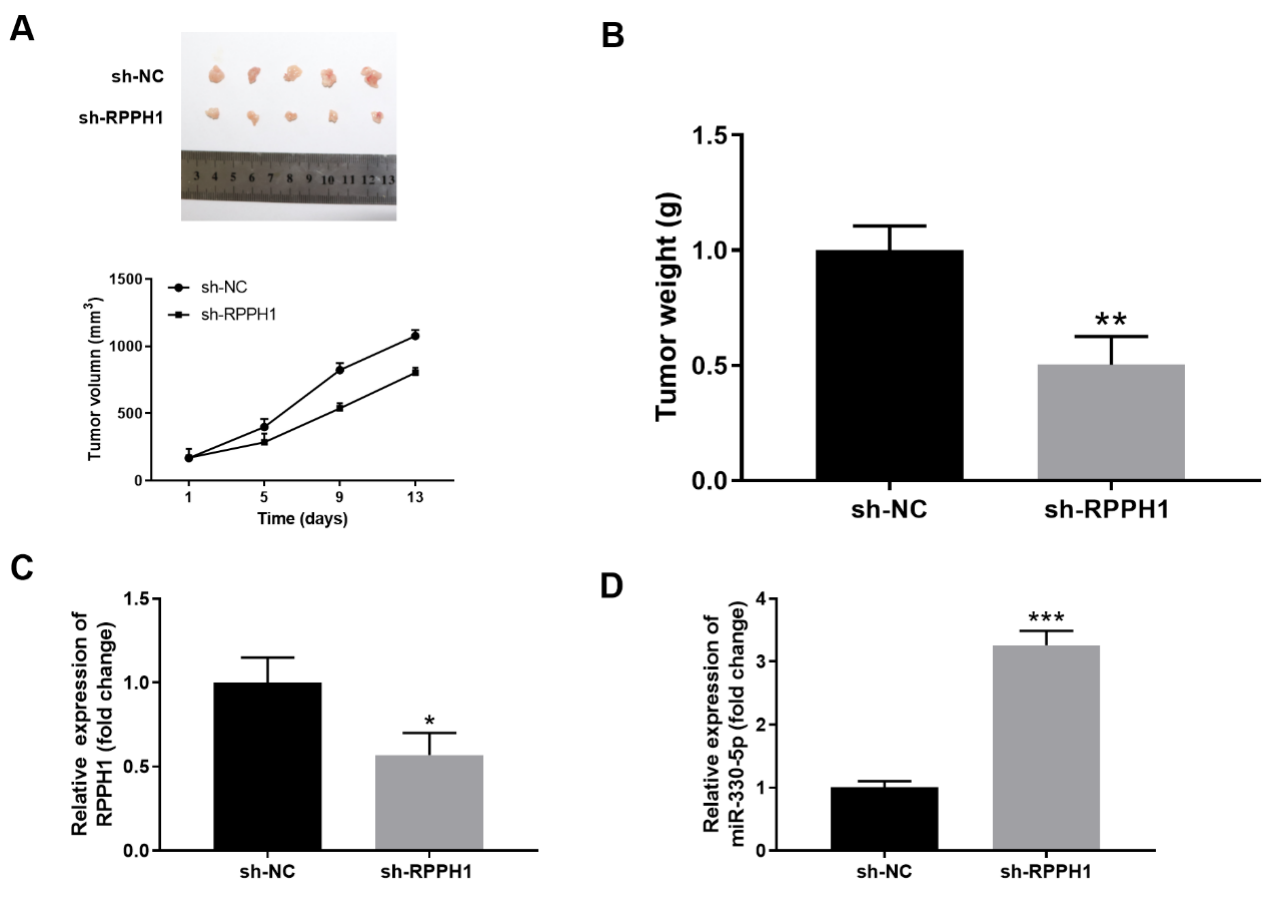
RPPH1 knockdown inhibited the *in vivo* growth of xenografted THP-1 tumor in nude mice. (A-D) Stable THP-1 cell line with knockdown of RPPH1 (sh-RPPH1) or control THP-1 cells (sh-NC) were injected subcutaneously into the right flank of each mouse (5×10^6 ^cells/mouse) to establish the xenograft model. Tumor volume was monitored twice per week, and tumor tissues were collected at two weeks after tumor inoculation. (A) The image of tumors and the tumor growth curves in the sh-NC and sh-RPPH1 groups. (B) The weight of tumors in the indicated groups. (C-D) The expression levels of RPPH1 (C) and miR-330-5p (D) in tumor tissues were determined by RT-qPCR. *n*=5 for each group; *P<0.05, compared with the sh-NC control group

To further investigate the role of RPPH1 in the progression of AML *in vivo*, we inoculated sh-NC lentivirus infected control stable THP-1 cells and sh-RPPH1 lentivirus infected stable THP-1 cells to the flank of nude mice by subcutaneous injection. As shown in Figure 5A(Fig. 5), RPPH1 knockdown resulted in significantly retarded growth of xenografted THP-1 cells, and the volumes of tumors in the sh-RPPH1 group were significantly smaller than that in the sh-NC group. Consistently, the mass of tumors in the sh-RPPH1 group was markedly reduced at two weeks after tumor inoculation (Figure 5B(Fig. 5)). In addition, we found that the tumor tissues from the sh-RPPH1 group had significantly decreased expression of RPPH1 (Figure 5C(Fig. 5)) and increased expression of miR-330-5p (Figure 5D(Fig. 5)) when compared with that from the sh-NC group. Taken together, these results implied the pro-tumor functions of RPPH1 and anti-tumor functions of miR-330-5p *in vivo*.

## Discussion

AML is highly prevalent in adults with high relapse rates, low overall survival rates and poor prognosis (Dohner et al., 2015[8]; Prada-Arismendy et al., 2017[22]). It is in urgent need to find out more effective biomarkers of AML to strengthen its diagnosis and treatment. Aberrant expression of lncRNAs has been described in many types of cancers (Ling et al., 2015[17]), and their functional involvement in the pathogenesis of AML is continuously being unraveled (Nobili et al., 2016[21]; Zebisch et al., 2016[31]). In this study, we explored the potential roles of RPPH1 in human AML cells and the impacts of RPPH1 expression on survival of patients with AML. We found that lentivirus-mediated interference of RPPH1 could effectively inhibit the proliferation, invasion and migration of AML cells, which was partially dependent on the up-regulated expression of miR-330-5p, one of the targets of RPPH1. Thus, we identified an RPPH1-miR-330-5p axis in the pathogenesis of human AML that might be used as a biomarker for AML diagnosis and treatment.

The present study demonstrated that RPPH1 was highly expressed in the blood cells of AML patients and human AML cell lines. It seems that RPPH1 acts as a carcinogenic lncRNA, as its elevated expression was found to be associated with poor overall survival of patients suffering from AML. Consistently, RPPH1 expression was also found to be involved in the progression of breast cancers. In paired clinical samples, breast cancer tissues had up-regulated expression of RPPH1 in comparison to the adjacent normal tissues. While RPPH1 over-expression promoted cell cycle and proliferation and increased colony formation of human breast cancer cell lines MCF-7 and MDA-MB-231, knockdown of RPPH1 significantly inhibited cell proliferation in both MCF-7 and MDA-MB-231 cells (Zhang and Tang, 2017[32]). Similarly, in our study, RPPH1 in human AML cells also exerted the pro-proliferative functions, as its silencing significantly inhibited AML cell growth. Moreover, lentivirus-mediated interference of RPPH1 was further demonstrated to be able to suppress the tumor formation in nude mice inoculated with either human breast cancer MCF-7 cells or AML THP-1 cells. Taken together, RPPH1 more likely functions as an oncogene in human diseases rather than just a well-known RNA subunit of RNase P involved in regulating tRNA (transfer ribonucleic acid) maturation, and plays important roles in the biological and pathological processes of breast cancer and AML.

A previous study demonstrated that the pathway of RPPH1/miR-330-5p/CDC42 is involved in the compensatory behavior of brain neurons to combat synaptic loss during the pathogenesis of Alzheimer's disease. In mouse cortexes and hippocampi, Rpph1 regulated miR-330-5p expression, and their interaction was substantiated by the dual luciferase reporter assay (Cai et al., 2017[5]). Consistently, our study also demonstrated that miR-330-5p was one of the targets of RPPH1 in human AML, and its expression was down-regulated in human AML cells in comparison to the control bone marrow stromal cells. Since miR-330-5p-specific inhibitor could reverse the retarded growth of AML cells with knockdown of RPPH1, miR-330-5p is more likely to function as a tumor suppressor. Indeed, in HL-60 cells, miR-330-5p has been revealed to negatively regulate the expression of T cell immunoglobulin and mucine domain (TIM)-3, a specific surface marker for leukemic stem cells (Fooladinezhad et al., 2016[11]). In addition, several studies have also identified the important role of miR-330-5p in other human cancers. For example, miR-330-5p negatively regulates integrin α5 (ITGA5) expression in colorectal cancer and glioblastoma, and its expression suppresses disease progression (Feng et al., 2017[9]; Yoo et al., 2016[30]). Moreover, miR-330-5p modulates tyrosinase and PDIA3 expression, which can result in inhibition of cell proliferation and invasion in cutaneous malignant melanoma (Su et al., 2016[25]). In addition, miR-330-5p was identified as a putative modulator of neoadjuvant chemoradiotherapy sensitivity in esophageal adenocarcinoma and in non-small cell lung cancer (Bibby et al., 2015[4]; Kong et al., 2017[15]). Collectively, our results and other results suggest that miR-330-5p is a tumor suppressor gene in both solid tumors and leukemic malignancies like AML.

As a member of the Rho GTPase family, CDC42 modulates actin dynamics and spinogenesis, and it is also a target of miR-330 in breast cancer and colorectal cancer (Bellot et al., 2014[2]; Jeyapalan et al., 2011[13]). Recently, CDC42 was unveiled to control AML cell polarity and division asymmetry, and represents a useful target to alter leukemia-initiating cell fate for differentiation therapy. Moreover, inducible CDC42 suppression in primary human AML cells blocks leukemia progression in a xenograft model (Mizukawa et al., 2017[20]). This suggests that a novel axis of RPPH1/miR-330-5p/CDC42 might also exist in human AML, and further experimental validation is required to substantiate it. It is worth noting that miR-330-5p inhibition can not fully reverse the anti-tumor effects of RPPH1 knockdown in AML cells. This might be due to the inadequate inhibition of miR-330-5p by transient transfection of the inhibitor, or that other downstream targets of RPPH1 rather than miR-330-5p also contribute to the roles of RPPH1 in AML progression. Therefore, further investigations on screening potential targets of RPPH1 are needed to fully elucidate the functions of RPPH1 in AML pathogenesis. 

The current investigation has several strengths and limitations. Its major strength is its novelty; to the best of our knowledge, this is the first report on unveiling the critical role of the lncRNA RPPH1 in AML pathogenesis. The current work also provides a feasible framework for validating AML progression related candidate lncRNA genes to better facilitate the diagnosis and implementation of more effective therapy of AML patients. One limitation of the current study is its sample size of XX patients and single data source when evaluating the impacts of RPPH1 expression on patient survival. In addition, more work on validating the therapeutic effects of *in vivo* knockdown of RPPH1 in animals is warranted to substantiate the potential of the RPPH1/miR-330-5p axis-targeted therapy in AML patients.

## Conclusion

We found that the lncRNA RPPH1 was significantly up-regulated in PBMCs of AML patients and human AML cell lines, and its higher expression correlated with worse overall survival of AML patients. Knockdown of RPPH1 markedly decreased the proliferation, invasion and migration ability of AML cell lines THP-1 and HL-60 *in vitro*, which was at least dependent on the expression of miR-330-5p, a direct target of RPPH1. Our *in vivo* xenograft mice experiments also demonstrated that down-regulation of RPPH1 significantly reduced THP-1 tumor growth. Our study improves the understanding of lncRNA functions in AML pathogenesis, and suggests that the RPPH1/miR-330-5p axis is a potential target for AML treatments.

## Acknowledgements

This work was supported by the National Natural Science Foundation of China (No. 8153000580, No.81270597).

## Conflict of interests

The authors declare that they have no competing interests.

## Authors’ contributions

LB, AL performed the statistical analyses, evaluated the results and drafted the paper. WG, LB, AL and YX participated in the conception and design of the study. LJ, XR and QL contributed to the enrollment of the patients and follow up. XM, PY contributed to analyzing data. All authors have read and approved the final manuscript.

## Supplementary Material

Supplementary material
