# Supplementary material for: Long non-coding RNA RPPH1 promotes the proliferation, invasion and migration of human acute myeloid leukemia cells through down-regulating miR-330-5p expression
Source: EXCLI J. 2019 Sep 11;18:824–37. doi: 10.17179/excli2019-1686 (PMC6806202; doi:10.17179/excli2019-1686)
Supplement: Supplementary material [file EXCLI-18-824-s-001.pdf]

EXCLI Journal 2019;18:824-837 – ISSN 1611-2156
Received: July 29, 2019, accepted: August 29, 2019, published: September 11, 2019

Supplementary material to:

LONG NON-CODING RNA RPPH1 PROMOTES THE PROLIFERATION, INVASION AND MIGRATION OF HUMAN ACUTE MYELOID LEUKEMIA CELLS THROUGH DOWN-REGULATING MIR-330-5P EXPRESSION

Bo Lei, Aili He, Yinxia Chen, Xingmei Cao, Pengyu Zhang, Jie Liu, Xiaorong Ma, Lu Qian, Wanggang Zhang*

Department of Hematology, second Affiliated Hospital of Xi'an Jiaotong University, 157 Xiwu Road, Xi'an, Shaanxi, China

* **Corresponding author:** Wanggang Zhang, PhD, Department of Hematology, second Affiliated Hospital of Xi'an Jiaotong University, 157 Xiwu Road, Xi'an, 710004, China, E-mail: zhangwanggang2003@yahoo.com

http://dx.doi.org/10.17179/excli2019-1686

This is an Open Access article distributed under the terms of the Creative Commons Attribution License (http://creativecommons.org/licenses/by/4.0/).

Supplementary Table 1: The primers used in the study

	Forward	Reverse
**RPPH1**	5’ -GAGCTGAGTGCGTCCTGTC-3’	5’ - TCAGGGAGAGCCCTGTTAGG- 3’
**miR-330-5p**	5'-ACACTCCAGCTGGGTCTCTGGGCCTGTGTC-3'	5'-CTCAACTGGTGTCGTGGAGTCGGCAATTCAGTTG AGTCCTAAGA-3'
**miR-326**	5'-TCATCTGTCTGTTGGGCTGG-3'	5'-TAAATCTGCCTCGGGACTGG-3'
**miR-328-3p**	5'-CGGGCCTGGCCCTCTCTGCC-3'	5'-CAGCCACAAAAGAGCACAAT-3'
**GAPDH**	5'-CCACATCGCTCAGACACCAT-3'	5'-CCAGGCGCCCAATACG-3'
**U6**	5'-ATTGGAACGATACAGAGAAGATT-3'	5'-GGAACGCTTCACGAATTTG-3'
